# Correction: Rapid DNA replication origin licensing protects stem cell pluripotency

**DOI:** 10.7554/eLife.49040

**Published:** 2019-06-05

**Authors:** Jacob Peter Matson, Raluca Dumitru, Philip Coryell, Ryan M Baxley, Weili Chen, Kirk Twaroski, Beau R Webber, Jakub Tolar, Anja-Katrin Bielinsky, Jeremy E Purvis, Jeanette Gowen Cook

Matson JP, Dumitru R, Coryell P, Baxley RM, Chen W, Twaroski K, Webber BR, Tolar J, Bielinsky A-K, Purvis JE, Cook JG. 2017. Rapid DNA replication origin licensing protects stem cell pluripotency. *eLife* **6**:e30473. doi: 10.7554/eLife.30473.

Published 17, November 2017

It has come to our attention that we made a typographical error in reporting a catalog number in this article related to the analytical flow cytometry method. The donkey anti-mouse 488 secondary antibody from Jackson Immunoresearch was originally listed incorrectly as catalog number 715-545-140, whereas the *correct* catalog number is 715-545-150. The article has been corrected accordingly. We apologize for the error.

